# Beyond antibiotics: phage-encoded lysins against Gram-negative pathogens

**DOI:** 10.3389/fmicb.2023.1170418

**Published:** 2023-09-08

**Authors:** Sanket Shah, Ritam Das, Bhakti Chavan, Urmi Bajpai, Sarmad Hanif, Syed Ahmed

**Affiliations:** ^1^Techinvention Lifecare Private Limited, Mumbai, India; ^2^Department of Biomedical Science, Acharya Narendra Dev College, University of Delhi, New Delhi, India

**Keywords:** phage-encoded lysins, antimicrobial resistance, Gram-negative bacteria, *Acinetobacter baumannii*, *Pseudomonas aeruginosa*, *Enterobacteriaceae*

## Abstract

Antibiotics remain the frontline agents for treating deadly bacterial pathogens. However, the indiscriminate use of these valuable agents has led to an alarming rise in AMR. The antibiotic pipeline is insufficient to tackle the AMR threat, especially with respect to the WHO critical category of priority Gram-negative pathogens, which have become a serious problem as nosocomial and community infections and pose a threat globally. The AMR pandemic requires solutions that provide novel antibacterial agents that are not only effective but against which bacteria are less likely to gain resistance. In this regard, natural or engineered phage-encoded lysins (enzybiotics) armed with numerous features represent an attractive alternative to the currently available antibiotics. Several lysins have exhibited promising efficacy and safety against Gram-positive pathogens, with some in late stages of clinical development and some commercially available. However, in the case of Gram-negative bacteria, the outer membrane acts as a formidable barrier; hence, lysins are often used in combination with OMPs or engineered to overcome the outer membrane barrier. In this review, we have briefly explained AMR and the initiatives taken by different organizations globally to tackle the AMR threat at different levels. We bring forth the promising potential and challenges of lysins, focusing on the WHO critical category of priority Gram-negative bacteria and lysins under investigation for these pathogens, along with the challenges associated with developing them as therapeutics within the existing regulatory framework.

## Introduction

Antibiotics are seen as ‘wonder drugs’ and, undoubtedly, are considered a blessing to human civilization in combating microbes and infections and saving millions of lives (Zaman et al., 2017). However, a gradual decline in antibiotic discovery and development from the time it peaked in the mid-1950s and the evolution of drug resistance in many pathogens owing to the indiscriminate use of these valuable agents has led to the alarming rise of antimicrobial resistance (AMR) (Hutchings et al., 2019). It represents one of the leading public health threats of the 21^st^ century, exacerbated by the overuse and misuse of antibiotics worldwide. The growing AMR crisis, temporarily overshadowed by the severe acute respiratory syndrome coronavirus 2 (SARS-CoV-2) pandemic, continues to severely affect global health and the economy (Llor and Bjerrum, 2014; Ghosh et al., 2021). It has been associated with severe infections, complications, longer hospital stays, and increased mortality. It is responsible for causing 700,000 deaths annually (Pokharel et al., 2020). In 2019, 4.95 million people were associated with drug-resistant bacterial infections, and 1.27 million deaths were directly caused by AMR (Antimicrobial Resistance, 2022). It is predicted that more than 10 million people will die annually due to AMR infections by 2050 (Ghose and Euler, 2020). Low- and middle-income countries (LMICs) remain at the epicentre of the AMR menace, and the O’Neill report has estimated that up to 90% of the 10 million projected deaths associated with AMR will occur in these countries (Singh et al., 2021). The SARS-CoV-2 pandemic has further intensified the AMR crisis due to the increased use of antibiotics to treat coronavirus 2019 (COVID-19) patients. It is likely to have caused more COVID-19 deaths, as secondary bacterial infections can worsen the prognosis of severely and critically ill patients (PAHO, 2021). There is an increasing recognition of the relationships between human and animal health, plant production, food safety, and environmental sectors in both the evolution and solution of the AMR problem. It is, therefore, essential to take the ‘One Health’ approach to effectively address the AMR issue (WHO, 2021). There are a number of organizations globally that are spearheading the battle to combat the threat posed by AMR (Table 1).

**Table 1:** Organizations working towards addressing the AMR threat.

Organization	Description
World Health Organization (WHO)	The global human health sector response to antimicrobial resistance (AMR) is led by the WHO, which collaborates closely with countries as they prioritize, implement, and evaluate their interventions. The AMR response is based on four strategic priority areas: (i) stepping up leadership for AMR response; (ii) driving public health impact in every country to address AMR; (iii) R&D for better access to quality AMR prevention and care; and (iv) monitoring the AMR burden and global AMR response (WHO, 2022).
Centre for Disease Control (CDC)	The CDC leads the public health response of the United States of America (USA) to combat antibiotic resistance. It addresses the AMR threat through collaboration with other federal agencies, state and local health departments, patients, public health partners, and the private sector. The CDC has invested in more than 330 innovative antibiotic resistance projects from 2016 to 2020, spanning more than 30 countries, to curb the spread of resistance globally (CDC, 2022).
Combating Antibiotic-Resistant Bacteria Biopharmaceutical Accelerator (CARB-X)	CARB-X is a global non-profit partnership dedicated to accelerating antibacterial research to tackle the threat of drug-resistant bacteria. It is funded by a global consortium of governments and foundations, focusing mainly on the WHO and CDC priority list of pathogens. The CARB-X portfolio represents the world’s most scientifically diverse early-development pipeline of new antibiotics, vaccines, rapid diagnostics, and other products to prevent, diagnose, and treat life-threatening bacterial infections (CARB-X, 2023).
AMR-Global	AMR-Global is a public-private partnership (PPP) that brings together experts from science, business, policy, and society in the fight against AMR. The organization works towards equitable access to effective and affordable antimicrobial solutions for all. Through innovations, the partnership works on improving infection prevention and control, access to improved diagnostics, antimicrobial stewardship, and effective antimicrobial drugs and vaccines tested in real-world settings globally (AMR-Global, 2022).
World Bank	The World Bank is financing 56 projects in 35 countries to strengthen and develop agricultural, health, water, and sanitation systems, which are critical in preventing the emergence and spread of resistance. Its financing and policy dialogue also provides governments with technical assistance and implementation support for AMR-related investments. It collaborates with international organizations, donors, and country partners to support awareness and understanding of critical issues relating to AMR through reports, training, seminars, and international convenings (World Bank, 2021).
Antimicrobial Resistance Surveillance and Research Network (AMRSN) - Indian Council of Medical Research (ICMR)	The ICMR, India, launched the AMRSN in 2013, which is responsible for generating data on drug-resistant infections and AMR patterns prevalent in the country. The data is being used as a template to devise guidelines, educate, and connect the members of the antimicrobial community. The agency provides newer insights into the molecular mechanisms of resistance, the clonality of drug-resistant pathogens, and the transmission dynamics to enable a better and more in-depth understanding of AMR in the Indian context. To strengthen the preventive measures and reduce antibiotic misuse, ICMR’s nationwide antimicrobial stewardship programme (AMSP) emphasizes on the judicious use of antimicrobials and improving diagnostic stewardship and infection control practices (AMRSN-ICMR, 2019).
Wellcome Trust	Wellcome Trust has been collaborating with the private sector and philanthropic partners to develop the $1 billion AMR Action Fund, which aims to bridge the funding gaps and technical barriers faced by antibiotic developers during later-stage clinical development and ensure the availability of new treatments for drug-resistant infections for the patients who need them the most. The AMR Action Fund aims to bring two to four new antibiotics to market in the next decade and support biotech companies to advance promising antibiotic candidates through the later stages of clinical trials and approvals (Wellcome, 2021).

Excessive and irresponsible antibiotic usage has led to the emergence of multi-drug-resistant (MDR) pathogens, which are associated with increased mortality, morbidity, prolonged hospitalizations, and a significant burden on the healthcare system (Paterson et al., 2016; Hong et al., 2022). MDR infections caused by Gram-negative bacteria are widely recognized as one of the most significant areas of unmet medical need due to the limited treatment options available and the slow pace with respect to the development of new antimicrobial therapeutics (Ghose and Euler, 2020). In particular, Gram-negative bacteria, such as carbapenem-resistant *Acinetobacter baumannii* (*A. baumannii*) and *Pseudomonas aeruginosa* (*P. aeruginosa*), and carbapenem-resistant and third-generation cephalosporin-resistant *Enterobacteriaceae*, are becoming a problem, especially in nosocomial infections (Hong et al., 2022). These pathogens pose a serious threat globally and are described as critical - highest level of concern among the World Health Organization’s (WHO) list of priority resistant bacteria for 2016–17 (Bassetti and Garau, 2021). The clinical pipeline of antibiotics is insufficient to tackle the threat posed by AMR, especially with respect to the WHO critical priority pathogens. Out of 42 new therapeutics that target the priority pathogens, only two of them belonging to the same class (siderophore-cephalosporins) target the critical priority pathogens (Gutierrez and Briers, 2021). The WHO has called for global action on AMR, which has encouraged several actions such as: (a) prevention and control actions in healthcare facilities; (b) antimicrobial stewardship programmes; (c) reducing antibiotic usage in livestock production and the environment; and (d) quest for suitable alternatives to the currently used antibiotics, especially for MDR pathogens having a global impact (Vazquez et al., 2021).

Bacteriophage (phage)-derived lytic proteins, viz., lysins, are fast emerging as protein-antibiotic candidates to mitigate the AMR crisis. Although phages have long been used for treating bacterial infections, lysins are relatively new (Murray et al., 2021). These phage-encoded lysins, termed ‘enzybiotics’, are armed with numerous properties and represent one of the most advanced classes of antibacterials under clinical investigation, having a novel mode of action based on peptidoglycan (PG) degradation. Lysins have shown success in killing Gram-positive bacteria. However, the presence of an outer membrane (OM) in Gram-negative bacteria acts as a barrier not just for the antibiotics but also for the lysins (Gutierrez and Briers, 2021).

**Figure 1:**
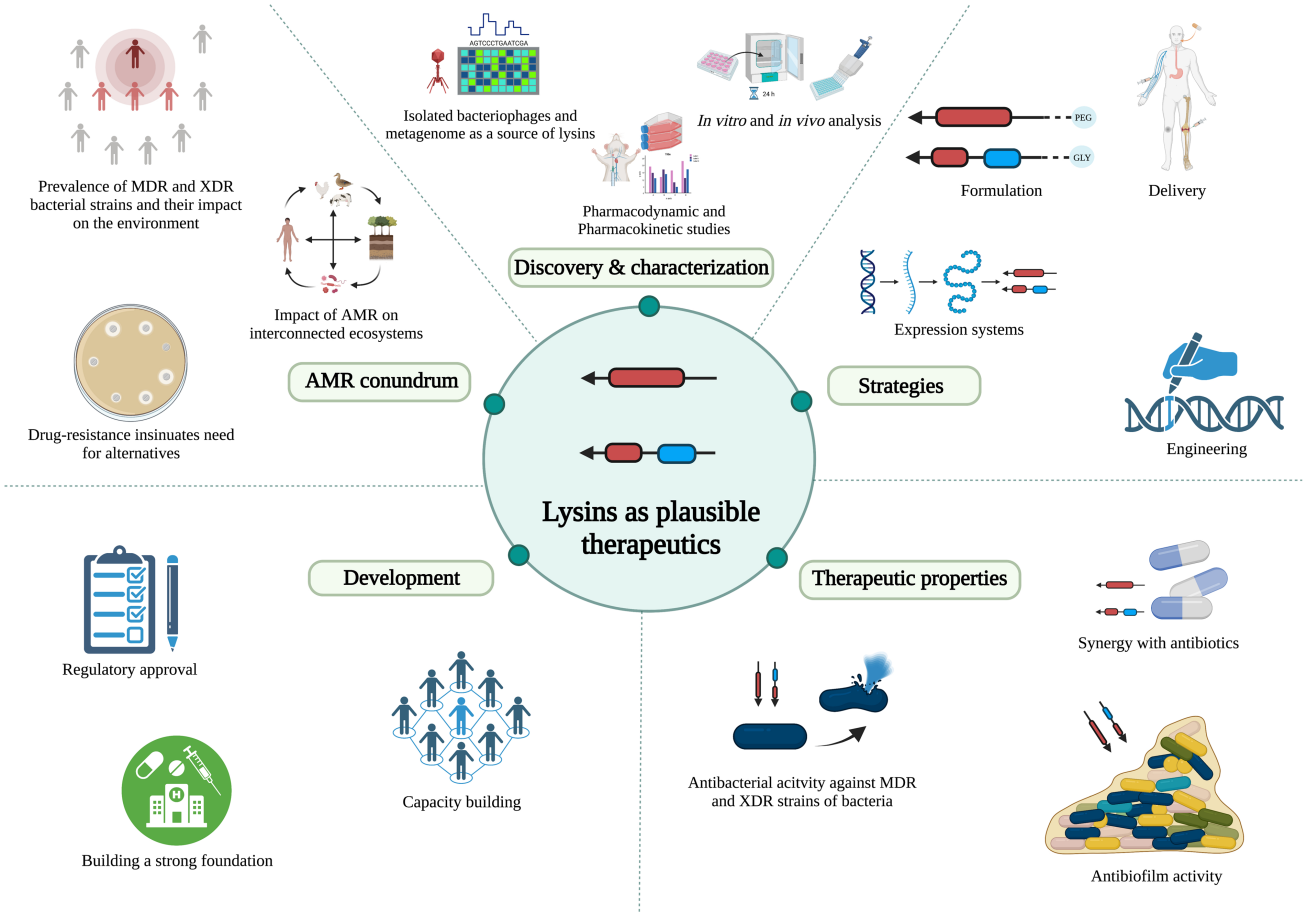
Schematic representation of lysin’s promising potential and development, focusing on Gram-negative bacteria as the targets. *AMR: antimicrobial resistance; MDR: multi-drug-resistant; XDR: extensively drug-resistant.

In this review, we bring forth the promising potential of lysin enzymes, focusing on the WHO critical category of priority Gram-negative bacteria as targets, and discuss the merits, mechanism of action, lysins under investigation for these pathogens, and the challenges associated with developing lysins as therapeutics within the existing regulatory framework (Figure 1).

## Lysins as ‘enzybiotics’

Phages infecting bacterial cells have evolved in two ways to release their progenies. While filamentous phages are extruded from the cells continuously without killing the host, non-filamentous phages encode for enzymes called (endo) lysins, which cleave the bacterial cell wall, inducing lysis of the host (Gondil et al., 2020). Without a signal sequence, these enzymes translocate to the periplasmic space using small hydrophobic phage-encoded proteins called holins (Hermoso et al., 2007). Many *in vitro* studies have been reported on several lysins, and a few have reached preclinical and clinical human trials (Jun et al., 2014; Kim et al., 2018; Raz et al., 2019). No adverse side effects during topical or intravenous applications were observed in these trials.

## Strategies to identify novel lysin sequences

Viruses that target and replicate inside a bacterial host are known as phages (Das and Bajpai, 2022; Das et al., 2023). They require escape strategies to exit the bacterium to disseminate their progenies. The lytic system of the phage, which constitutes lysin enzymes that target PG, essentially weakens bacterial cell walls, causing lysis (Fischetti, 2008). Phages can be an intrinsic source of several antibacterial proteins, and among those, phage-encoded lysins have been demonstrated as efficient bactericidal proteins (Nelson et al., 2001; Fenton et al., 2010; Gondil et al., 2020; Das et al., 2022). Considering the efficiency of killing and their activity against drug-susceptible and drug-resistant bacteria, lysins can be a potential game-changer as alternative adjuncts to antibiotics and in managing AMR crisis. Lysins can be derived from isolated phages (Gondil et al., 2020) and uncultured viral genomes and characterized (Fernandez-Ruiz et al., 2018). Recent metagenomic analysis of the viral ‘dark matter’ revealed insights into the phage genomes that were inaccessible through standard molecular techniques (Fernandez-Ruiz et al., 2018). Discovering novel lysin candidates showing efficient antibacterial activity and developing them as therapeutics is now increasingly pursued. Screening of environmental samples, such as water, soil, and sewage, has demonstrated the abundance and diversity of the virome, which is untapped but is a potential source for several enzymes of biotechnological value, including the phage-derived lysin enzymes. The PhaLP open portal^1^ has an entry of 17,566 lysins. This comprehensive lysin database consists of information on physicochemical properties, modular diversity, host specificity, and clusters of lysins, which is easily accessible and can serve as a blueprint for protein engineering (Criel et al., 2021).

To investigate viral metagenome as a source of lysins, Fernández-Ruiz et al. discovered thousands of putative lysin sequences in uncultured phage genomes, of which several were predicted to be effective against pathogenic bacteria. This study identified about 46 orthologous groups of lysins consisting of 62 domains, including 26 catalytic domains, 12 cell wall-binding domains (CBDs), and the remaining 24 domains of unknown function (Fernandez-Ruiz et al., 2018). A study by Vázquez et al. compiled a database of around two thousand lysin sequences and uncovered the diversity among the domains, which can help design novel future antimicrobials (Vazquez et al., 2021). These studies demonstrate how uncultured phage genomes and culture-independent techniques could add to the known repertoire of lysins.

## Structure and types of lysins

The origin and target of the lysins determine their structure. Lysins against Gram-positive bacteria are generally modular, containing an enzymatically active domain (EAD) and a CBD with a molecular weight of 25–40 kDa (Fischetti, 2008). The EAD is the catalytic domain of the lysin and cleaves various bonds in the PG, and CBD binds to specific molecules in the PG, thus facilitating the activity of the EAD (Briers et al., 2014b). These domains are associated with linker regions, which are short and flexible.

Lysins active against Gram-negative bacteria have a molecular weight in the 15–20 kD range. The domain organization is mostly globular (containing only the EAD), though exceptions are also reported (Fischetti, 2008; Abdelrahman et al., 2021). Recently discovered lysins, KZ144 and EL188, of phages infecting *P. aeruginosa* are broad-spectrum lysins with a modular structure. KZ144 has a PG binding domain and an EAD (transglycosylase and lysozyme-like domain). Lysin EL188 has two PG binding regions and a soluble lytic transglycosylase domain (Fischetti, 2008). Signal-arrest-release lysins (ERA103, Lyz103, and LyzP1) against Gram-negative bacteria have also been discovered. These were found to be localized in the periplasmic space before the initiation of cell wall lysis and can therefore work holin-independent (Abdelrahman et al., 2021).

**Figure 2:**
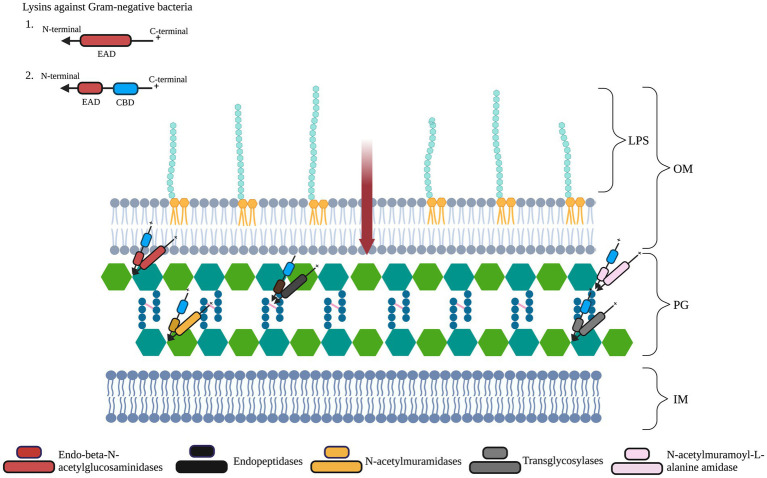
Schematic representation of lysin targets in Gram-negative bacterial cells’ peptidoglycan layer (PG). The PG layer of the cell wall is composed of repeating units of sugars N-acetylglucosamine (GlucNAc) and N-acetylmuramic acid (MurNAc). The PG consists of tetrapeptide bridges cross-linked by pentapeptide bridges (pink lines). The inner membrane is represented as IM. Gram-negative lysins have an enzymatically active domain (EAD, represented in red ellipses) and/or a cell wall-binding domain (CBD, represented in blue ellipses). On exogenous application, lysins with a cationic C-terminal region can transverse (red arrow) the lipopolysaccharide (LPS)-containing outer membrane (OM). Differently colored EADs represent the bonds they cleave. Figure adapted from Das et al. (2022).

These studies and the databases highlight the diversity in the structural organization of lysins. This diversity persists when we look into the types of EAD they contain. Broadly, there are three classes of six different EADs: (1) Endopeptidases: Lysins such as D-alanyl-glycyl endopeptidase (CHAP) and D-glutamate endopeptidase (VANY), which are involved in the cleavage of stem peptide-interpeptide bridge or within interpeptide bridges of two amino acids (aa). (2) Glycosidases: Lysins of this class lyse the bonds that link alternating polymeric molecules of N-acetylmuramic acids (MurNAc) and N-acetylglucosamines (GlcNAc) present in the PG. These include N-acetyl-β-D-muramidases, lytic transglycosylases, and N-acetyl-β-D-glucosidases. (3) Amidases: The amide bond present between L-alanine and MurNAc of the PG is cleaved by enzymes of this class, *viz.*, N-acetylmuramoyl-L-alanine amidase (Lu et al., 2021; Das et al., 2022; Figure 2).

## Therapeutic, pharmacokinetic, and pharmacodynamic properties of lysins

**Figure 3:**
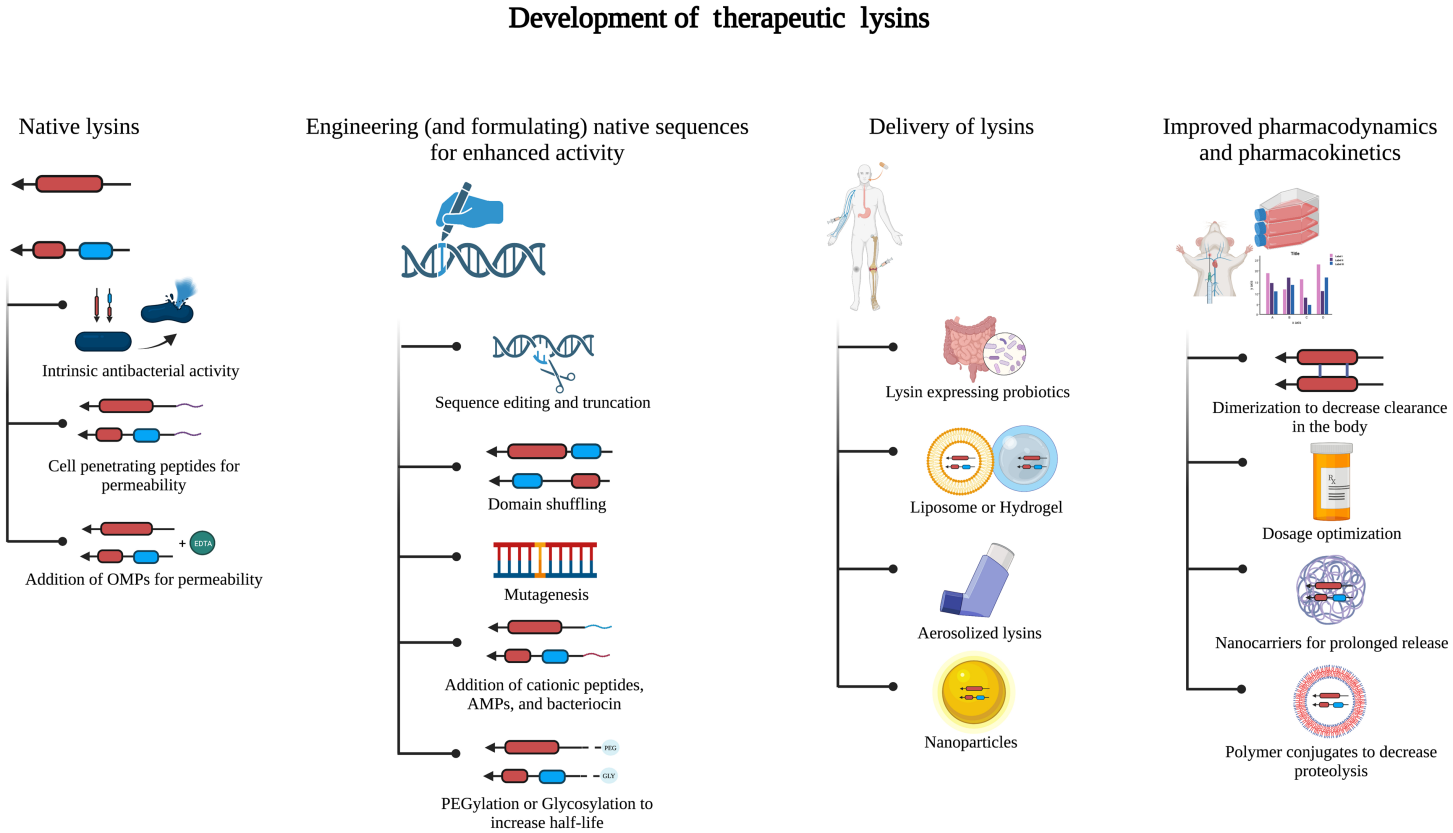
Development of therapeutic lysins. Native lysins without intrinsic antibacterial activity can be modified by adding cationic, amphipathic, or antimicrobial peptides (AMPs) or using outer membrane permeabilizers (OMPs). Lysins are engineered and formulated to enhance their stability and antibacterial activity. This has been done by sequence editing or truncation, domain shuffling, and PEGylation or glycosylation. Delivery of engineered lysins can be as nanoparticles or by using liposomes or hydrogels, aerosolization, or probiotics expressing the lysin of interest. Processes such as dimerization, nanocarriers, and polymer conjugates assist in improved pharmacodynamics and pharmacokinetics.

Lysins have several advantages over other antimicrobial agents, including phages (Kaur et al., 2020a,b; Figure 3). Lysins in clinical studies have not yet been shown to negatively impact the healthy microflora that is attributed to the high specificity of lysins to their host. One of the expected downsides of using lysins as therapeutics is their potential to evoke humoral immune responses. Though most studies have not reported an immune response, except for a few pre-clinical studies where phage-specific antibodies were found, neutralizing antibodies have not been observed so far (Fenton et al., 2010; Zhang et al., 2016). An effective way to lower the immune response is by PEGylation of lysins, which reduces their binding to antibodies, as done in lysostaphin (Bastos et al., 2010). Though PEGylation can also reduce enzyme activity, this is compensated by enhanced kinetics and a decrease in the blocking effect of antibodies (Figure 3).

One of the most appealing aspects of lysins as antibiotics is the remote propensity of the target bacterium to gain resistance against them, even after several generations of usage in low dosages (Loeffler et al., 2001; Schuch et al., 2002). This is because lysins target PG that is essential for bacterial cell integrity and survival. Also, these enzymes recognize specific molecules present in the PG that are essential for cell viability (Fenton et al., 2010).

While many reports on lysins have been published in the last two decades, studies on prospective medications’ pharmacokinetics and pharmacodynamics properties are limited. The absorption, distribution, metabolism, excretion, and toxicity (ADMET) score plays a vital role in determining the efficacy of a chemical drug during its discovery and development (Guan et al., 2019). For developing lysins as protein drugs, their bioavailability, distribution, toxicity, and similar parameters are also critical for obtaining approval to understand better whether lysins can progress to preclinical and clinical studies (Murray et al., 2021; Figure 3).

## Synergistic effect of lysins with antibiotics

Another vital aspect of lysins is their ability to have an enhanced effect on pathogens by synergistically acting with other antimicrobial agents. The synergy between the two antibacterial agents can increase bactericidal potency and endurance. The narrow-spectrum antibacterial activity of lysins limits the undesirable killing of commensal bacteria, and the biofilm-disrupting potential of lysins increases antibiotic exposure and the killing of pathogens. Hence, using lysins in combination with antibiotics can be overall more efficacious (Schuch et al., 2014).

Antibiotics and lysins used together to target Gram-negative bacteria have shown encouraging results. The synergy of LysABP-01with seven routinely used antibiotics was evaluated against one MDR and four extremely drug-resistant *A. baumannii* strains. *In vitro*, a synergetic activity was identified between LysABP-01 and colistin, with a fractional inhibitory concentration (FIC) ratio of 0.156–0.188. The minimum inhibitory concentrations (MICs) of LysABP-01 and colistin were lowered to 32-fold and 8-fold, respectively (Thummeepak et al., 2016). Colistin, a last-resort antibiotic, treats infections caused by MDR Gram-negative bacteria. Due to its nephrotoxicity and neurotoxicity, it is required to be used at a low dose (Nation and Li, 2009), and this can possibly be achieved by using a combination of colistin with lysins. The synergistic effects of lysin ElyA1 and colistin in targeting a variety of MDR Gram-negative bacteria, including *A. baumannii*, *P. aeruginosa*, and *Klebsiella pneumoniae* (*K. pneumonia*), have recently been confirmed in both *in vitro* and *in vivo* settings (Blasco et al., 2020).

## Effect of lysins on bacterial biofilms

**Figure 4:**
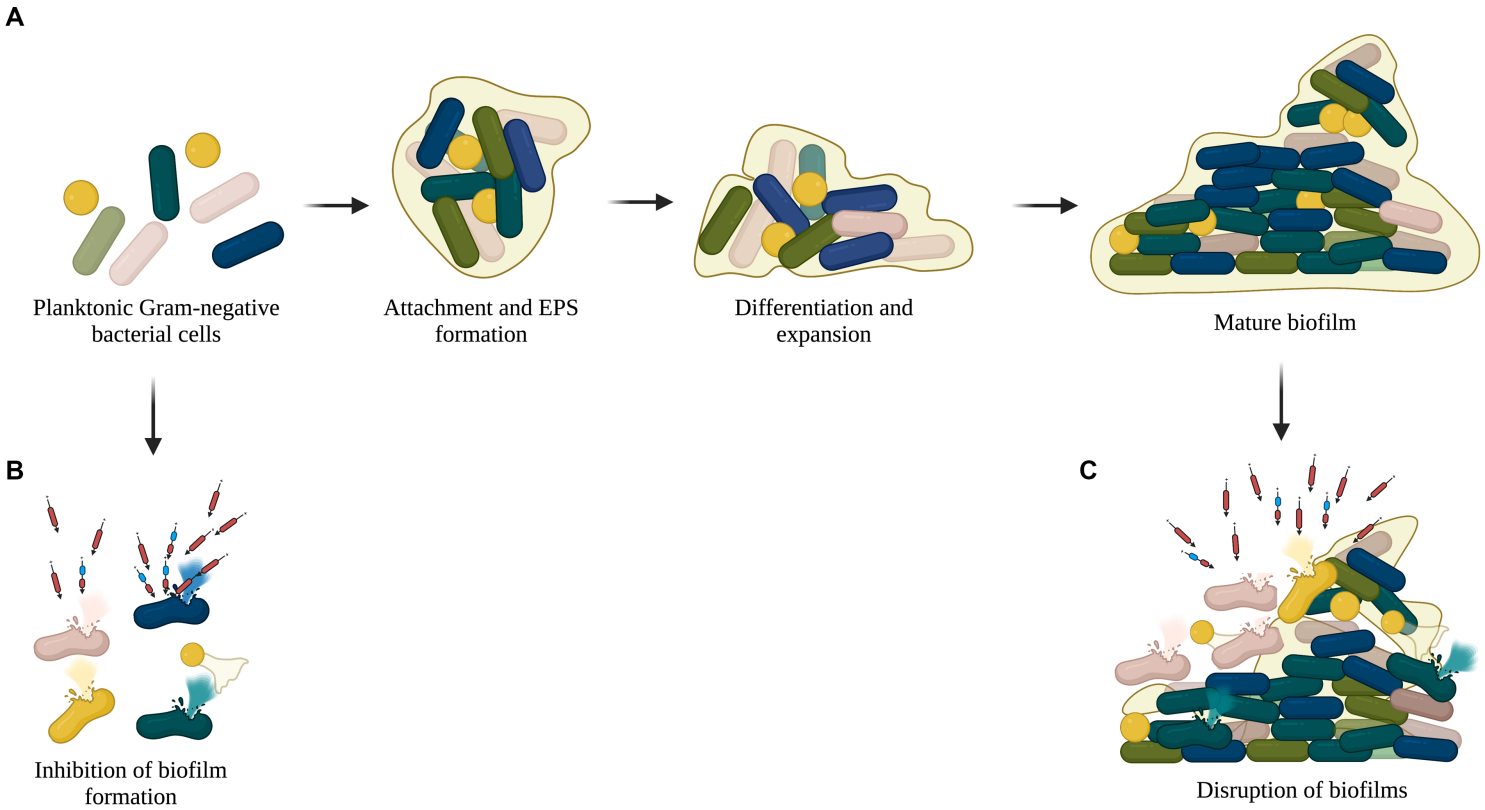
Antibiofilm activity of recombinant lysins. **(A)** Planktonic cells adhere to abiotic or biotic surfaces and form biofilm with a complex matrix composed of polysaccharides, lipids, nucleic acids, and proteins. **(B)** Lysins lyse planktonic cells and inhibit biofilm formation. **(C)** Pre-formed or mature biofilms disrupted by lysins.

Biofilms are a consortium of pathogenic microorganisms that adhere to abiotic and biotic surfaces and are associated with infections caused by the use of implants, drains, and catheters (Lusiak-Szelachowska et al., 2020). Self-produced polysaccharides, lipids, nucleic acids, and proteins form a matrix around the bacterial population, rendering antibiotics less susceptible or inefficacious (Pires et al., 2017). With the change in the exopolysaccharide matrix and bacterial population, the dynamics of the biofilm may differ with time, which can further decrease susceptibility to antibiotics, causing resistant strains to emerge (Mah and O'Toole, 2001). Several studies have explored the antibiofilm activity of lysins, which show their efficiency in inhibiting biofilm formation and disrupting pre-formed biofilms (Diez-Martinez et al., 2015; Chu et al., 2022; Figure 4). Taken together, these properties of lysins make a strong case for their application as one of the potential solutions to the AMR crisis by serving as an alternative or complement to the available antibiotics.

## Examples of lysins under investigation against WHO’s critical category of priority Gram-negative pathogens

There are a number of lysins that have been investigated against WHO’s critical category of priority Gram-negative pathogens, as shown in Table 2.

**Table 2:** Lysins under investigation for WHO critical category of priority pathogens.

Sr. No.	Lysin	Enzymatic activity	Modifications	Outer membrane permeabilizers	Target bacteria	References
1	EndoT5	Peptidase	–	Polymyxin B, gramicidin D, poly-l-lysine, chlorhexidine, and miramistin	*E. coli*	Shavrina et al. (2016)
l2	Lysep3-D8	Lysozyme (Muramidase)	Fusing *B. amyloliquefaciens* bacteriophage lysin binding domain D8 to the C-terminal region of Lysep3	–	*E. coli*, *P. aeruginosa*, *A. baumannii*, and *Streptococcus* strain	Lv et al. (2015) and Wang et al. (2017)
3	Colicin-Lysep3	Lysozyme (Muramidase)	N-terminal translocation region and central receptor binding region of colicin A fused with Lysep3	–	*E. coli*	Lv et al. (2015) and Yan et al. (2017)
4	Lysep3 + aa	Lysozyme (Muramidase)	Fusing 5,10, and 15 cationic amino acid polypeptide and a polypeptide including both cationic and hydrophobic aa to the C-terminus of Lysep3 to obtain four fusion lysins (5aa, 10aa, 15aa, and mix).	EDTA and citric acid	*E. coli*	Lv et al. (2015) and Ma et al. (2017)
5	Lysin KP27	Endopeptidase	–	–	*K. pneumoniae*, *P. aeruginosa*, *S.* Typhimurium, and *E. coli*	Maciejewska et al. (2017a)
6	APgp15	Lysozyme (Muramidase)	–	Chloroform	*E. coli*, *K. pneumoniae*, *P. aeruginosa*, *B. enocepacia*, and *S.* Typhimurium	Maciejewska et al. (2017b)
7	Ply3a03 and PlyPa03	Lysozyme (Muramidase)	–	–	*P. aeruginosa*	Raz et al. (2019)
8	gp144	Transglycosylase	–	–	*P. aeruginosa*, *E. coli*, *S. aureus*, and *B. cereus*	Paradis-Bleau et al. (2007)
9	EL88	Transglycosylase	–	EDTA, citric acid, poly-l-lysine, and polymyxin B nonapeptide (PMBN)	*P. aeruginosa*	Briers et al. (2008) and Briers et al. (2011)
10	Art-175	Transglycosylase	Fusing *P. aeruginosa* lysin KZ144 to a sheep myeloid peptide (SMAP-29) having antimicrobial properties	EDTA	*E. coli*, *P. aeruginosa*, and *A. baumannii*	Briers et al. (2009), Briers et al. (2014a), Defraine et al. (2016), and Schirmeier et al. (2018)
11	PyS2-GN4	Transglycosylase	Fusing *P. aeruginosa* bacteriocin pyocin S2 (PyS2) domains I to III to the *P. aeruginosa* phage PAJU2 lysin GN4	–	*P. aeruginosa*	Heselpoth et al. (2019)
12	LysPA26	Lysozyme (Muramidase)	–	–	*E. coli*, *P. aeruginosa*, *A. baumannii,* and *K. pneumoniae*	Guo et al. (2017), Murray et al. (2021)
13	LoGT-008	Lysozyme (Glycoside hydrolase)	Polycationic nonapeptide fused to the N terminal of PVP-SE1gp146	EDTA	*P. aeruginosa* and *A. baumannii*	Briers et al. (2014b) and Abdelrahman et al. (2021)
14	LysAB2	Muramidase	–	–	*A. baumannii*, *E. coli*, and *S. aureus*	Lai et al. (2011)
15	LysAB2 P3	Muramidase	–	–	*A. baumannii*	Peng et al. (2017)
16	PlyF307	Muramidase	–	–	*A. baumannii*	Lood et al. (2015)
17	PlyE146	Muramidase	–	–	*E coli*, *P. aeruginosa*, and *A. baumannii*	Larpin et al. (2018)
18	ElyA1	Muramidase	–	–	*P. aeruginosa*, *A. baumannii*, and *K. pneumoniae*	Blasco et al. (2020) and Lai et al. (2020)
19	P307SQ-8C	Muramidase	Eight aa (SQSRESQC) from the hepatitis B virus fused to the C-terminal of P307		*A. baumannii*	Thandar et al. (2016)
20	LysABP-01	Muramidase	–	–	*A. baumannii*	Thummeepak et al. (2016)
21	PlyAB1	Glycoside hydrolase	–	–	*A. baumannii*	Huang et al. (2014)
22	Abgp46	Muramidase	–	EDTA, citric acid, and malic acid	*A. baumannii*, *P. aeruginosa*, and *S.* Typhimurium	Oliveira et al. (2016)
23	Ply6A3	Muramidase	–	–	*A. baumannii*, *S. aureus*, *K. pneumonia*, *P. aeruginosa*, and *E. coli*	Wu et al. (2018)

### Pseudomonas aeruginosa

Ply3a03 and PlyPa91 have exhibited bactericidal properties against various *P. aeruginosa* clinical strains, including biofilm-embedded strains. Using minimum biofilm eradication concentration (MBEC) biofilm inoculator 96-well plate system, treatment with PlyPa03 resulted in the complete elimination of *P. aeruginosa* biofilms at all concentrations tested down to 0.375 mg/mL. PlyPa91 resulted in >1-log colony-forming unit (CFU) drop at 0.375 mg/mL, >2-log CFU drop at 0.75 mg/mL, and complete biofilm elimination at 1.5 mg/mL. Ply3a03 and PlyPa91were effective in the treatment of a *P. aeruginosa* skin infection in a mouse model, and PlyPa91 protected mice in a lung infection model. Treatment of the infected skin with 300 μg PlyPa03 resulted in a > 2-log mean reduction in the bacterial load, and treatment with 100 μg PlyPa91 resulted in a 1-log reduction in bacterial counts. Mice treated with 1.8 mg/mL PlyPa91 in two intranasal instillations resulted in a significant delay in death, with 20% of the mice surviving at day 10. Mice treated with one intranasal (1.8 mg/mL) and one intratracheal (1.8 mg/mL) instillation demonstrated a further reduction in the death rate, with 70% of the mice surviving at day 10 (Raz et al., 2019). Adding purified lysin gp144 to purified PG from four Gram-negative bacteria caused a rapid OD_600nm_ decrease of PG in a dose-dependent manner. Lysin gp144 (100 mM) hydrolyzed PG of *P. aeruginosa* in 2 minutes (min) and *E. coli* in 25 min. A lag of >60 min was observed for the PGs of *Bacillus cereus* (*B. cereus*) and *Staphylococcus aureus* (*S. aureus*), and significant hydrolysis was detected only after 120 min (Paradis-Bleau et al., 2007). Antibacterial assays demonstrated that incubation of 10^6^ *P. aeruginosa* cells/mL with 10 mmol ethylenediaminetetraacetic acid (EDTA) and 50 μg/mL lysin EL188 led to a strain-dependent inactivation between 3.01 ± 0.17 and 4.27 ± 0.11 log units in 30 min. Increasing the EL188 concentration to 250 μg/mL further increased the inactivation of antibiotic-resistant strain Br667 (4.07 ± 0.09 log units) (Briers et al., 2011).

To address the challenges arising from the co-administration of lysins and outer membrane permeabilizers (OMPs), substantial efforts have been directed toward the engineering of the lysins to enhance their permeability to the OM of Gram-negative bacteria (Vazquez et al., 2018). One such example is the development of Artilysins that involve the fusion of lysins to an OM destabilizing peptide that exhibits activity against Gram-positive and Gram-negative pathogens (Ghose and Euler, 2020). Art-175 is a broad-spectrum artilysin constructed by fusing *P. aeruginosa* lysin KZ144 to a sheep myeloid peptide (SMAP-29), which also serves as a potent antimicrobial peptide (AMP) (Briers et al., 2009; Defraine et al., 2016; Schirmeier et al., 2018; Vazquez et al., 2018; Ghose and Euler, 2020). *In vitro,* bactericidal assays have demonstrated that Art-175 exhibited a bactericidal effect against *P. aeruginosa*, *A. baumannii*, and *Escherichia coli* (*E. coli*) (Briers et al., 2014b; Defraine et al., 2016; Schirmeier et al., 2018). Art-175 (3 μM) breached the OM and killed *P. aeruginosa*, including MDR strains, in a rapid and efficient (5 log units) manner (Briers et al., 2014a). Using the microdilution method, MICs of Art-175 ranged between 4 to 20 μg/mL, showing the broad inhibitory activity of Art-175 against different MDR strains of *A. baumannii*. The addition of EDTA (200 μM) further reduced the MIC of Art-175 and improved its antibacterial effect for all strains under investigation (ranging from ≤4 to 10 μg/mL) (Defraine et al., 2016). The susceptibility of the *E. coli* isolates to Art-175 was evaluated using the broth microdilution method. Art-175 inhibited all colistin-resistant isolates, and their corresponding average MIC values (9.5 ± 3.0 μg/mL, ranging from 6.3 to 15.8 μg/mL) were not significantly different from those of the colistin-susceptible isolates (11.3 ± 2.7 μg/mL, ranging from 7.5 to 16.3 μg/mL) (Schirmeier et al., 2018).

PyS2-GN4 is a lysocin engineered by fusing *P. aeruginosa* bacteriocin pyocin S2 (PyS2) domains I to III to the *P. aeruginosa* phage PAJU2 lysin GN4. The bactericidal activity of PyS2-GN4 was assayed in iron-deficient casamino acids (CAA) medium as a function of antimicrobial concentration and time. During 12 hours (h) of incubation, PyS2-GN4 (0.1 to 100 μg/mL) killed *P. aeruginosa* bacteria at similar rates, resulting in nearly 2-, 3-, and 4-log reductions in bacterial viability at 2, 4, and 12 h, respectively. PyS2-GN4 and tobramycin disrupted biofilm biomass at ≥0.16 μg/mL. It demonstrated MIC values of ≤4 μg/mL for *P. aeruginosa* reference strain PAO1 and 452, 453, and MDR-M-3 clinical isolates. In a murine model of bacteremia, when mice were treated with 2.5, 5, 12.5, and 25 mg/kg lysocin intraperitoneally, 73, 80, 93, and 100%, respectively, were protected from death (Heselpoth et al., 2019).

LysPA26 is another pH- and heat-stable phage-encoded lysin that exhibits intrinsic lytic activity against MDR *P. aeruginosa* without utilizing OMPs (Murray et al., 2021). LysPA26 (0.5 mg/mL) killed about 4 log units bacteria in 30 min when incubated with 10^8^ exponential *P. aeruginosa* D204 cells without EDTA. When the challenged cells were pretreated with EDTA (1 mM), more than 1 log cells were killed by LysPA26, indicating EDTA enhanced the antibacterial activity of LysPA26. It also efficiently destroyed existing *P. aeruginosa* biofilm cells in a dose-dependent manner. The optical density (OD_600nm_) staining of the biofilms was significantly reduced upon the addition of LysPA26 (upto 50 μg). Approximately 1–2 log in the number of viable counts of biofilm cells were disrupted by LysPA26 (100 μg). LysPA26 (0.5 mg/mL) also exhibited antibacterial activity against the tested MDR clinical samples of other Gram-negative bacteria, *viz.*, *A. baumannii, K. pneumoniae, P. aeruginosa*, and *E. coli,* as demonstrated through a decrease in the viable bacterial numbers (Guo et al., 2017). The ability to target an array of MDR Gram-negative bacteria makes such lysins promising candidates for future lysin therapy (Murray et al., 2021).

The activity of LoGT-008, a novel lysin, has been evaluated by Briers et al. with MIC against *P. aeruginosa* and *A. baumannii* responsible for skin infections (Briers et al., 2014b; Abdelrahman et al., 2021). LoGT-008 demonstrated MICs of 4 and 8 μg/mL against *P. aeruginosa* and *A. baumannii*, respectively. These values corresponded to 0.15 and 0.30 μM, which were in the same range as ciprofloxacin (0.5 μM), a commonly used first-line antibiotic for many Gram-negative infections. In a human neonatal foreskin keratinocyte model infected with *P. aeruginosa* PA14 (10^5^ or 10^7^ CFU/mL), LoGT-008 fully protected (100%) the keratinocyte monolayer from the cytotoxic effects of PA14 infection with 10^5^ CFU/mL. The protection was associated with a drastic decrease in the bacterial load (Briers et al., 2014b).

### Acinetobacter baumannii

LysAB2 is a lysin isolated from *A. baumannii* phage ɸAB2. MDR *A. baumannii* (BCRC 80276), *E. coli* (Top 10), and methicillin-resistant *Staphylococcus aureus* (MRSA) (BCRC 80277) were examined for their susceptibility to LysAB2. The viabilities of *A. baumannii* and *E. coli* dropped to less than 1% following the incubation with LysAB2 (500 μg/mL) for 1 h. The viability of MRSA (BCRC 80277) retained 18% after incubation with LysAB2 for 1 h (Lai et al., 2011). The AMPs with bactericidal properties were extracted from the C-terminal of LysAB2 and subsequently investigated *in vitro* and *in vivo* against MDR strains of *A. baumannii.* In *A. baumannii* mouse model of ascites (inoculated intraperitoneally with 2 × 10^7^ CFU of *A. baumannii* ATCC 17978), LysAB2 P3 (100 μM/mouse, 3.7 mg/kg) administered intraperitoneally significantly reduced the bacterial load (~1,500 CFU/mL) by 13-fold compared with that of PBS control mice. In *A. baumannii* mouse model of bacteremia (inoculated intraperitoneally with 5 × 108 CFU of *A. baumannii* ATCC 17978), 32 μM/mouse (1.2 mg/kg) and 200 μM/mouse (7.5 mg/kg) of LysAB2 P3 given intraperitoneally after 1 h and 3 h, respectively, reduced the bacterial load by 27-fold and 6-fold, respectively, compared with those in PBS (10 mL/kg) control mice (Peng et al., 2017).

PlyF307 is a lysin isolated from an environmental strain of *A. baumannii*. Its C-terminal exhibits a high positive net charge, and its target site is the OM and inner membrane of *A. baumannii*. PlyF307 (100 μg/mL) exhibits *in vitro* bactericidal activity against 13 clinical isolates (>5-log-unit decrease in 2 h) (Lood et al., 2015). The efficacy of PlyF307 has also been demonstrated against *A. baumannii* biofilms formed on polyvinyl catheters and *in vivo* in a murine *A. baumannii* bacteremia model. Treatment with PlyF307 (250 μg) resulted in approximately 1.6-log-unit reduction in the biofilm on the catheters. Also, scanning electron microscopy (SEM) showed substantial destruction of the extracellular polymer matrix of the biofilm. After intraperitoneal injection of *A. baumannii* (10^8^ CFU) in mice to develop systemic bloodstream infection, a single therapeutic dose (1 mg) administered intraperitoneally saved 50% of the bacteremic mice. In contrast, all the mice in the control group died within 24 h (Lood et al., 2015).

PlyE146, encoded by *E. coli* prophage, is shown to have activity against *E coli, P. aeruginosa*, and *A. baumannii*. PlyE146 (400 μg/mL) exhibited *in vitro* bactericidal activity against *E. coli* K12 (3.6 log_10_ CFU/mL decrease) after 2 h of incubation at 37°C without NaCl and pH 6.0. Under these conditions, PlyE146 also displayed antimicrobial activity towards other *E. coli*, *P. aeruginosa* (3 to 3.8 log_10_ CFU/mL decrease), and *A. baumannii* (4.9 to >5 log_10_ CFU/mL decrease) strains (Larpin et al., 2018).

The synergistic effects (*in vitro* and *in vivo*) between lysin ElyA1 and colistin have been demonstrated in MDR *A. baumannii*, *P. aeruginosa*, and *K. pneumoniae* by Blasco et al. There was a 4-fold reduction in colistin’s MIC when combined with ElyA1 (Blasco et al., 2020; Lai et al., 2020). In a murine skin model infected with a clinical strain of *A. baumannii* GMA001, the cell counts were significantly lower in the colistin combination treatments (with both doses of ElyA1, *viz.*, 50 μg and 350 μg) than in the buffer control. The cell counts in the 350 μg ElyA1 plus colistin treatment were also significantly lower than in the colistin control. In a mouse lung model infected with *A. baumannii* GMA001, lung CFU counts were significantly lower in the mice treated with the combination of colistin and ElyA1 than in the mice treated with buffer or with colistin (Blasco et al., 2020).

P307SQ-8C lysin is a construct made by fusing the positive C-terminal of PlyF307 lysin and eight aa from the hepatitis B virus core protein. *A. baumannii* treated with P307SQ-8C (50 μg/mL) resulted in a 3.2-log reduction after 5 min, with a continued reduction in bacterial count up to 5 logs at 120 min. At a concentration of 200 μg, it reduced the bacterial burden by ∼2 logs in a murine model of *A. baumannii* skin infection in 2 h (Thandar et al., 2016).

LysABP-01 from *A. baumannii* bacteriophage ØABP-01 has a globular structure consisting of a lysozyme-like (N-acetyl-β-D-muramidase) catalytic domain. Using bacterial growth inhibition assays, LysABP-01 prevented the growth of *A. baumannii* with a MIC of 20 mM. The combination of LysABP-01 plus colistin demonstrated elevated antibacterial activity (nearly 100% of growth inhibition rate). Using checkerboard assay, the FIC index value for the combination of LysABP-01 and colistin was 0.188, which indicated synergism (Thummeepak et al., 2016). *A. baumannii* AB1 was lysed effectively by the purified PlyAB1 protein. The OD_600nm_ of the reaction between AB1 and PlyAB1 decreased from 1.05 to 0.2 within 30 min. The viable bacterial counts decreased from 1.4 × 10^7^ CFU/mL to 4.1 × 10^6^ CFU/mL (82% reduction) in 30 min with 100 μg/mL of PlyAB1. In the lytic activity assay, PlyAB1 digested all 48 hospital-derived pandrug-resistant (PDR) isolates of *A. baumannii*, which was evident when the OD_600nm_ of the PDR isolates dropped significantly when purified PlyAB1 was incubated with them (Huang et al., 2014). When ABgp46 (2 μM) was used in the presence of OMPs, *viz.*, citric acid (3.65 mM) and malic acid (4.55 mM), >5 log reduction was observed in *A. baumannii* strain #2, whereas *P. aeruginosa* and *Salmonella enterica serovar* Typhimurium (*S.* Typhimurium) showed a greater than 4 log reduction (Oliveira et al., 2016). Using spot assay, lysin Ply6A3 (1 mg/mL) degraded 141/200 clinical MDR *A. baumannii* isolates and 21/40 PDR *A. baumannii* isolates. In addition, it was also able to degrade *Enterococci, S. aureus*, *K. pneumonia*, *P. aeruginosa*, and *E. coli*. The concentrations of the isolates under investigation were 10^9^ CFU/mL. In the *A. baumannii* sepsis mouse model (developed through intraperitoneal administration of 1 mL 10^9^ CFU/mL of AB32), the survival rate with Ply6A3 (2 mg/mL) was 70% higher as compared to the bacterial group (Wu et al., 2018).

### Enterobacteriaceae

#### Escherichia coli

EndoT5 is a PG hydrolase that belongs to the M15 family of peptidases, and *in vitro* studies have shown its ability to lyse *E. coli* strains in the presence of various OMPs, *viz.*, polymyxin, gramicidin D, poly-l-lysine, chlorhexidine, and miramistin. EndoT5, in combination with polymyxin B (0.4 μg/mL) or chlorhexidine (0.5 μg/mL), reduced the number of CFUs by five orders, and in combination with poly-l-lysine (80 μg/mL) by four orders, as compared to control (Shavrina et al., 2016). Lysep3 is a coliphage lysin isolated from *E. coli-*specific bacteriophage vB_EcoM-ep3, exhibiting *in vitro* bactericidal effect against *E. coli* and *P. aeruginosa* (Lv et al., 2015). Lysep3 cannot penetrate the OM of *E. coli*, so several modifications have been made to this lysin to breach the OM. The fusion of *Bacillus amyloliquefaciens* (*B. amyloliquefaciens*) bacteriophage lysin binding domain D8 to the C-terminal region of Lysep3 enhanced its bactericidal activity on exogenous application. Lysep3-D8 lysed Gram-negative and Gram-positive bacteria, whereas lysep3 and D8 had no impact on bacterial growth. The MIC of lysep3-D8 on *E. coli* was 60 μg/mL. Lysep3-D8 inhibited bacterial growth for up to 12 h at this concentration. It could lyse all 14 *E. coli* strains, 3 *P. aeruginosa* strains, 1 *A. baumannii* strain, and 1 *Streptococcus* strain under investigation. Lysep3-D8 demonstrated sufficient bactericidal effects on the 14 *E. coli* strains tested at 100 μg/mL (Wang et al., 2017; Murray et al., 2021). Other modifications in Lysep3 include fusing it with colicin A or adding positively charged amino acids or hydrophobic aa at the C-terminus (Ma et al., 2017; Yan et al., 2017, 2019). Colicin-Lysep3 lysed *E. coli* broadly *in vitro* and significantly reduced the number of *E. coli* in an intestinal infection mouse model. The *E. coli* BL21 (DE3)-green fluorescent protein (GFP) content in the forepart of the small intestine of mice fed Colicin-Lysep3 was 11% less as compared to the control group, 3% less in the posterior segment of the small intestine, and 3% less in the cecum (Yan et al., 2017). The C-terminus of the *E. coli* phage lysin Lysep3 was fused to different peptides containing 5–15 aa and a polypeptide including both cationic and hydrophobic aa to obtain four fusion lysins, *viz.*, 5aa, 10aa, 15aa, and mix. Lysep3 or fusion lysins (5aa, 10aa, 15aa, and mix) at 0.75 μg/μL were added to *E. coli* BL21 (DE3) (10^5^ CFU/mL) in medium along with the commonly used OMPs, *viz*., EDTA (0.5 mM) and citric acid (2 mM), to investigate its bactericidal activity. The results showed that the bactericidal activities of fusion lysins increased by 1.1–1.5 fold in the presence of EDTA compared to Lysep3. The bactericidal activity of mix and 5aa increased by 1.1-fold, 10aa activity increased by 1.2-fold, and 15aa activity increased by 1.3-fold. With citric acid, 5aa activity increased by 1.1-fold, mix and 10aa activity increased by 1.2-fold, and 15aa activity increased by 1.5-fold compared to Lysep3 (Ma et al., 2017).

#### Klebsiella pneumoniae

Lysins demonstrating lytic activity against *Klebsiella* have been isolated from phages K11, KP32, and KP27 (Vazquez et al., 2018). Lysin KP27 has exhibited bactericidal activity against MDR *K. pneumoniae*, whereas AP3gp15, a muralytic enzyme encoded on the *Burkholderia* AP3 phage, has broad *in vitro* antibacterial activity against several pathogens, viz., *Burkholderia cenocepacia* (*B. cenocepacia*), *E. coli*, *K. pneumoniae*, *P. aeruginosa*, and *S.* Typhimurium (Maciejewska et al., 2017a,b). Using turbidimetric assays, the PG degrading activity of KP27 has been demonstrated on various OM permeabilized Gram-negative species and strains. For *K. pneumoniae* ATCC 700603 and 486 isolates, a specific activity of 9580 and 27,360 U/mg, respectively, has been shown; and for *P. aeruginosa* PAO1, *S.* Typhimurium, and *E. coli*, a specific activity of 23,700, 17,790, and 17,230 U/mg was observed, respectively (Maciejewska et al., 2017a). The muralytic activity of AP3 lysin was assessed on permeabilized Gram-negative strains according to a standardized assay methodology. The AP3gp15 was two times more effective than the commercially available lysozyme. The enzyme showed consistent activity against all tested strains, viz., *E. coli* (ATCC 25922) (AP3 lysin: 14,120 U/mg, commercial lysozyme: 7400 U/mg)*, K. pneumoniae* (ATCC 700603) (AP3 lysin: 11,970 U/mg, commercial lysozyme: 6200 U/mg), *P. aeruginosa* PAO1 (ATCC 15692) (AP3 lysin: 14,710 U/mg, commercial lysozyme: 8250 U/mg), *B. cenocepacia* 7780 (AP3 lysin: 9000 U/mg, commercial lysozyme: 3600 U/mg), and *S.* Typhimurium (AP3 lysin: 12690 U/mg, commercial lysozyme: 6800 U/mg) (Maciejewska et al., 2017b).

## Expression systems for the production of recombinant lysins

From a pharmaceutical standpoint, it is essential to ensure a higher yield of recombinant proteins for therapeutic applications (Kumraj et al., 2022). The development of lysins as the next generation of protein antimicrobials insinuates its cost-effectiveness and stable mass production. However, yields could be hampered due to low expression or formation of inclusion bodies (IB) and the inability to recover the recombinant proteins from IB from the bacterial expression systems. Low yields can make commercial-scale production and processing cost prohibitive. Therefore, evaluating varied expression systems is an important aspect of the enhanced yield of lysins.

Studies have been conducted to improve the existing *E. coli-*based protein expression. Balaban et al. demonstrated the use of osmolytes, such as N-lauroylsarcosine, to increase the expression rate of *E. coli* C43 (DE3) from the existing 30–40% to about 54% (Balaban et al., 2022). Engineered strains of *E. coli* BL21 (DE3), *viz.*, BL21Star (DE3) that offers enhanced mRNA stability, BL21trxB (DE3) that promotes correct folding of proteins and formation of disulphide bonds, and BL21 (DE3) pLysS that lowers the leaky expression of the gene of interest, are examples of *E.coli* expression systems that have been developed and found superior to their counterparts (Jia and Jeon, 2016).

Expression hosts other than prokaryotes have also been tested for generating active lysins (Oey et al., 2009; Rosales-Mendoza et al., 2012; Roach et al., 2013; Stoffels et al., 2017; Kazanaviciute et al., 2018; Tripathi and Shrivastava, 2019; Ahmad et al., 2020; Srinivasan et al., 2020). Roach et al. explored *Saccharomyces cerevisiae* (*S. cerevisiae*) expression system for producing recombinant lysins active against *Lactobacillus* spp (Roach et al., 2013). *Pichia pastoris* (*P. pastoris*) X-33, a methylotrophic yeast, has also been studied as a system for lysin production due to its fast growth rate and high levels of recombinant protein expression. Vplys60, a lysin active against *Vibrio parahaemolyticus* (*V. parahaemolyticus*) MTCC-45, was expressed in *P. pastoris* and obtained in high yield. The purified Vplys60 was active with strong amidase and antibiofilm activity against *V. parahaemolyticus* in an *in vivo* set-up (Srinivasan et al., 2020).

The eukaryotic green unicellular alga, *Chlamydomonas reinhardtii* (*C. reinhardtii*), also offers several benefits for expressing recombinant proteins, *viz.*, low cost and high scalability. The chloroplast of *C. reinhardtii* enables defined insertion of transgenes and markedly enhanced expression compared to the nuclear genome, and therefore has been studied as a model organism for protein production (Rosales-Mendoza et al., 2012). Stoffels et al. used this system and demonstrated the expression of lysins, *viz.*, Cpl-1 and Pal. The total yield of the recombinant lysins expressed in the alga was quantified to be ~1.3 mg/g of algal dry weight, and the enzymes were also found to be active against *Streptococcus pneumoniae* (*S. pneumoniae*) (Stoffels et al., 2017).

Transgenic plants have also been demonstrated to be efficient in producing recombinant lysins. Kazanavičiūtė et al. expressed six lysins targeting *Clostridium perfringens* (*C. perfringens*) in *Nicotiana benthamiana* (*N. benthamiana*) using *Agrobacterium tumefaciens* (*A. tumefaciens*)-mediated transfer, and the recombinant proteins constituted approx. 30% of the total soluble fraction, and the purified lysins retained its antibacterial activity (Kazanaviciute et al., 2018). Another study by Oey et al. showed recombinant lysin to be approx. 70% of the total soluble proteins when expressed in the plastids of tobacco plants. The high yield of the plastid-expressed active lysin could be attributed to its resistance to the host proteases. The generated recombinant protein was also active and demonstrated sufficient antibacterial activity (Oey et al., 2009).

These studies highlight the importance of exploring different expression approaches to obtain high yields of active recombinant proteins, which can effectively contribute towards scalable and cost-effective therapeutic lysin production. In Table 3, we have summarized our comparative analysis of different expression systems for lysin production.

**Table 3:** Different expression systems and their features for recombinant lysin production.

Expression Systems	Feasibility	Scalability	Yield	Production time	Cost	References
*Escherichia* spp.	High	High	Low	Low	High	Tripathi and Shrivastava (2019) and Kumraj et al. (2022)
Yeast	High	High	Medium	Medium	Medium	Jia and Jeon (2016), Balaban et al. (2022), and Kumraj et al. (2022)
Microalgae	Medium	High	Low	Low	Medium	Rosales-Mendoza et al. (2012) and Stoffels et al. (2017)
Transgenic plants	Medium	High	High	Medium	High	Oey et al. (2009), Kazanaviciute et al. (2018), and Kumraj et al. (2022)

## Engineering and formulation strategies to improve the lytic activity of lysins

Engineering and formulation of lysins are the two key strategies used to increase the antimicrobial properties of lysins and their delivery. Most of the naturally occurring lysins show lytic activity against Gram-positive bacteria. In contrast, in Gram-negative bacteria, the OM prevents the access of lysins to the target PG. To overcome the OM barrier, various engineering and formulation strategies have been developed over the years (De Maesschalck et al., 2020; Figure 3). Lysins with modular structures provide a unique opportunity for protein engineering to alter bacteriolytic activity, specificity, solubility, and other physicochemical features (Haddad Kashani et al., 2018). Engineering strategies involve the truncation of a full-length enzyme, site-directed mutagenesis, or various fusions, *viz.*, EADs with CBDs of different lysins, virion-associated lysin with CBD of another lysin, lysin fusion with AMP, etc. (Love et al., 2018).

Formulation strategies comprise the utilization of various carrier systems. A study showed how the fusion of the cellulose-binding module (CBM) tag assisted in anchoring T4 lysozyme to a cellulosic gauze material used for wound treatment (Abouhmad et al., 2016). Formulation with OMP chelators, viz., EDTA, citric acid, malic acid, lactic acid, benzoic acid, or acetic acid, has been demonstrated to be effective in permeabilizing the OM of Gram-negative bacteria to lysins (Oliveira et al., 2016; Figure 3).

Similarly, nanoencapsulation can aid in improving therapeutic potential by not only protecting lysins from degradation but also by enabling their sustained release and potentially increasing their stability, shelf life, and therapeutic efficacy (Gondil and Chhibber, 2021). Another study showed the feasibility of alginate-chitosan nanoparticles as a medication delivery platform for the lysin LysMR-5 (Kaur et al., 2020a). The ability of liposomes to permeate bacterial membranes via membrane fusion is also a promising method to formulate and deliver lysins to the target Gram-negative bacteria. In this study, the authors demonstrated successful lysis of both *S.* Typhimurium and *E. coli* with lysin BSP16Lys-containing liposomes without membrane permeabilizer pretreatment (Bai et al., 2019). These examples suggest the importance of leveraging the available strategies for improving the prospects of lysins as an effective and sustainable therapeutics.

### Lysin development against Gram-negative bacteria

Over the years, several studies have produced chimeric lysins by domain shuffling, by addition of AMPs, and by fusion with other proteins, viz., bacteriocins, receptor binding domain, etc., to enhance their efficacy, stability, permeability, expand their host range, or enhance their specificity (Yan et al., 2019; Gerstmans et al., 2020; Zampara et al., 2020; Duyvejonck et al., 2021). Protein engineering of lysins can create a library of variants that can be screened using medium- to high-throughput assays to select the most effective lysins with the potential to be developed as therapeutics.

Chimeric lysins have shown promising results in *in vitro* experiments (Yang et al., 2014). Those generated by domain shuffling have shown improved activity. The chimeric lysin created by the fusion of the EAD of Ply187 with the CBD of phiNM3 could lyse clinical isolates of MRSA *in vitro* and *in vivo* experiments and was more efficient than the parental lysin and lysostaphin (Yang et al., 2017). Engineered lysins through platforms, viz., ‘Artilysins’ and VersaTile’, appear to be a productive and unique way to generate a large number of candidates for therapeutic applications and populate the preclinical pipeline (Briers et al., 2014a; Gerstmans et al., 2020). Another study demonstrated improved activity of chimeric variants of a *Salmonella* phage lysin when fused with novel antimicrobial peptides generated from leucocin A. The enhanced anti-*Salmonella typhi* (*S. typhi*) activity in infected chickens helped in their increased shelf life (Nie et al., 2021). Adding cationic and hydrophobic peptides to lysins is another efficient method to generate novel effective lysin candidates, such as Lysep3. It was modified by adding 5–15 cationic aa at its C-terminal. The chimeric lysins showed better activity against *E. coli* than the parent lysin (Ma et al., 2017). A similar study with hydrophobic aa (3–12) attached to Lysep3 showed the modified lysins to lyse *E. coli* effectively on external application (Yan et al., 2019).

## Delivery of lysins

Targeted delivery of lysins at the site of the bacterial infection is critical. Various routes of administration to the target site in human or animal models are intravenous, nasal, oral, intraperitoneal, intramammary, topical, subcutaneous, etc. In a pioneering study, Nelson et al. found the oral administration of a group C streptococcal phage lysin, PlyC, to eliminate the streptococcal burden from heavy colonized murine oral cavities of mice (Nelson et al., 2001). Since then, several studies on developing lysins with improved formulation strategies for clinical applications, outlining topical uses and systemic applications, have been reported (De Maesschalck et al., 2020). The examples include subcutaneous injection of LysP108 in combination with intravenous injection of vancomycin to treat MRSA-infected mice (Lu et al., 2021), intraperitoneal injection of LysGH15 in a mouse model of bacteremia caused by *Staphylococcus epidermidis* (*S. epidermidis*) B020 (Zhang et al., 2018), intravenous injection of LysSAP26 to *A. baumannii*-infected mice (Kim et al., 2020), and topical application of S25-3 in a mouse model of staphylococcal impetigo (Imanishi et al., 2019). Intramammary treatment with LysRODI in mice prevented the mastitis lesions and significantly reduced the bacterial virulent infection caused by *S. aureus* (Gutierrez et al., 2020).

## Regulatory aspects of therapeutic lysins

The guidelines for the regulatory approval of lysins as antibacterial agents should be framed for their use in animals and humans as per the mandate followed in each country. Since lysins are protein antimicrobials, regulatory approvals can be sought after examining the criteria for approving several recombinant protein-based products already available for therapeutic applications. A detailed characterization of the safety, efficacy, and bioavailability data must be generated to seek such approvals (Cooper et al., 2016).

Currently, three possible alternative pathways can be applied to lysins as therapeutics. These are classical licensing, adaptive licensing, and compassionate use. Classical licensing can be a suitable pathway for lysins since it is already in place for antibacterial medications and hence should be appropriate for phage-derived proteins. The challenges associated with classical licensing include the high cost associated with trial recruitment and conducting additional trials for reformulation. In the case of adaptive licensing, it is advantageous for a small population with varying degrees of complexity. This requires a significant amount of time and money, whereas, in the case of compassionate use, immediate clinical usage is followed by data that can be used to inform future work that requires less time and money. Still, this therapy is limited to a single patient and is not approved for use (Cooper et al., 2016).

### Regulatory guidelines for biologics in Europe and US

In Europe, the European Medicines Agency (EMA) is the key regulatory authority responsible for approving new drugs and therapeutics. The EMA oversees clinical trials, safety monitoring, manufacturing, production, and marketing of a new drug. The regulatory requirements are listed under Directive 2001/83/EC and Regulation 726/2004/EC (Iglesias-Lopez et al., 2019). In the United States of America (USA), the biologics license application (BLA) requests authorization to deliver a biologic product for introduction into interstate commerce (21 CFR 601.2). The BLA is governed by the regulations found in 21 CFR 600–680. The primary requirements for a BLA include information on the product and manufacturing process and preclinical studies (FDA, 2021).

### Regulatory guidelines for biologics in India

In India, biologics are regulated under the Drugs and Cosmetics Act of 1945 and are based on the regulations for producing, using, importing, and exporting microorganisms and genetically engineered organisms in 1989 (Natarajan and Janani, 2019). The Department of Biotechnology (DBT) and review committee on genetic manipulation (RCGM) monitor the preclinical evaluation of recombinant biologics. The Central Drug Standard Control Organization (CDSCO), under the control of the Drugs Controller General of India (DCGI), regulates the safety and effectiveness of drugs in the country. Its primary mandate is the approval of clinical trials, authorization for marketing and manufacturing of new medicines, and issuing a license to manufacture similar biologics in India (Natarajan and Janani, 2019).

Regardless of the approach selected, the overall cost of drug research and the low return on investment of antibacterial drugs will continue to be a major concern. The approval of phage-derived lysins as protein therapeutics can be sought under the existing processes because of their similarity in the mode of action to traditional antibiotics. The discovery and development of lysin candidates against different infectious organisms can streamline the regulatory approvals for their clinical trials and therapeutic applications (Cooper et al., 2016).

## Conclusion

Antibiotics represent the frontline agents for treating deadly bacterial pathogens that are associated with high mortality, morbidity, increased healthcare expenditure, prolonged hospital stays, and reduced livelihoods. However, the current clinical pipeline of antibiotics is insufficient to mitigate the threat posed by AMR, especially with respect to the WHO critical category of priority Gram-negative pathogens. In such a grim situation, the development of novel therapeutics needs to be accelerated, and the complex AMR issue must also be addressed from a ‘One Health’ perspective. The burgeoning levels of AMR requires solutions that provide novel antibacterial agents that are effective and the bacteria are less likely to gain resistance against them. In this regard, phage-encoded lysins (natural or engineered) represent an attractive alternative or adjunct to the currently available antibiotics. Lysins have exhibited the required efficacy and safety in Gram-positive pathogens, with some in the late stages of clinical development and some already commercially available. In the case of Gram-negative bacteria, modifications have been devised to overcome the OM barrier, and several lysins have shown promising results in *in vitro* and *in vivo* studies and reached the preclinical stage. However, there are concerns regarding the efficacy, safety, pharmacokinetics, pharmacodynamics, formulation, dosage, route of administration, and the regulatory approval that warrant further investigation. Sustained R&D efforts and continuous funding for research and preclinical and clinical studies can pave the way for establishing lysins as therapeutics before AMR causes irreversible damage to us.

## Author contributions

SS, RD, BC, SH, and SA were involved in the literature search, writing, and revising of the manuscript. UB critically reviewed and edited the manuscript. All authors contributed to the article and approved the submitted version.

## Funding

This study was funded by Techinvention Lifecare Private Limited.

## Conflict of interest

SS, RD, BC, and SH are employed by Techinvention Lifecare Private Limited.

The remaining authors declare that the research was conducted in the absence of any commercial or financial relationships that could be construed as a potential conflict of interest.

## Publisher’s note

All claims expressed in this article are solely those of the authors and do not necessarily represent those of their affiliated organizations, or those of the publisher, the editors and the reviewers. Any product that may be evaluated in this article, or claim that may be made by its manufacturer, is not guaranteed or endorsed by the publisher.

## Footnotes

^1^ https://www.phalp.org/
